# Ameliorative Effect of Active Principle Isolated from Seeds of *Eugenia jambolana* on Carbohydrate Metabolism in Experimental Diabetes

**DOI:** 10.1093/ecam/nep233

**Published:** 2011-02-20

**Authors:** Suman Bala Sharma, Reenu Rajpoot, Afreena Nasir, Krishna Madhava Prabhu, Pothapragada Suryanarayana Murthy

**Affiliations:** ^1^Department of Biochemistry, University College of Medical Sciences, University of Delhi, Delhi 110095, India; ^2^B-164, Sector 14, Noida, India

## Abstract

The aim of this study was to evaluate the antidiabetic activity of LH II purified from ethanolic seed extract of *Eugenia jambolana* in alloxan-induced mild diabetic (MD) and severely diabetic (SD) rabbits. Ethanolic extract upon chromatographic purification yielded partially purified hypoglycemic principle (SIII) which on further purification by sephadex LH 20 yielded pharmacological active compound LH II. Homogeneity of LH II was tested by HPLC. Phytochemical investigation of LH II by various structural spectra showed the presence of saturated fatty acid, Δ^5^ lipid and presence of sterol. LH II was administered orally at a dose of 10 mg kg^−1^ body weight to MD and SD. LH II resulted, significant fall in FBG at 90 min (21.2% MD: 28.6% SD), 7th day (35.6% MD) and 15th day (59.6% SD). Glycosylated hemoglobin was significantly decreased (50.5%) in SD after 15 days treatment (Tt). Plasma insulin levels were significantly increased (*P* <  .001). *In vitro* studies with pancreatic islets showed 3-fold increase in insulin levels as compared to untreated animals. LH II also showed extrapancreatic effect by significantly increasing (*P* <  .001) the activity of key enzymes of glycolysis and significantly decreasing (*P* <  .001) the activity of key enzymes of gluconeogenesis. Liver and muscle glycogen content were increased by 36.6 and 30% for MD, and 52 and 47% for SD, respectively. Thus, the present study demonstrates that LH II possesses potent antidiabetic activity and it is effective in both MD and SD rabbits.

## 1. Introduction

The world is facing an explosive increase in the incidence of diabetes mellitus. According to the World Health Organization (WHO) estimates, the number of adults with diabetes in the world will rise from 135 million in 1995 to 300 million in the year 2025 [1]. India is the diabetic capital of the world, predicted to have 57.2 million diabetic population by the year 2025 [2].

A balance between glucose production and its utilization is necessary to maintain normal blood glucose levels. Diabetes is characterized by elevated production and low utilization of glucose [3]. A number of changes in several enzymes present in the liver and other tissues are known to occur in diabetes mellitus, for example, activity of hepatic glucokinase is markedly decreased and activity of glucose-6-phosphatase is almost doubled [4]. This imbalance results in constant hyperglycemia in the diabetic state. The chronic hyperglycemia of diabetes is associated with long-term dysfunction and damage to various organs. Hence, there is a need to search for a medication for lowering glucose as well as modify the alteration of key enzymes involved in carbohydrate metabolism.

Despite the great strides that have been made in the understanding and management of diabetes, the disease and disease-related complications are increasing unabated [5]. Though different types of oral hypoglycemic agents are available along with insulin for the treatment of diabetes mellitus, healers heavily relied upon medicinal plants and herbs to treat diabetes. Actually more than 1200 plants have been described to be experimentally or ethnopharmacologically used in the treatment of diabetes [6–15]. Based on the WHO recommendations, hypoglycemic agents of plant origin are important and warrant attention [16].

This article describes the study of active principle purified from ethanolic seed extract of *Eugenia jambolana* (Myrtaceae, common name: Black plum/Black berry in English and Jamun/Jambul in Hindi). *Eugenia jambolana* is a large tree found in all forests over the greater part of India from the sub-Himalayan tract to extreme south. It is also found in Thailand and Philippines. Fruits are oval to elliptical 1.5–3.5 cm long, dark purple or nearly black, luscious, fleshy and edible [17].

The antihyperglycemic activity of seeds of *E. jambolana* is well documented [18–27]. Aqueous and ethanolic extracts of seeds administered orally to experimental animals and to human adults at various dose levels were found to be active [20, 28]. Not only antihyperglycemic activity, but hypolipidemic activity is also reported in our earlier studies [23]. However, very few scientific studies are available about purification/isolation of active principle from ethanolic seed extract of *E. jambolana*. In this article, we report the purification of active principle from ethanolic seed extract of *E. jambolana* and study the effect of purified active principle on carbohydrate metabolism, in mild diabetic (MD) and severely diabetic (SD), which is so far not reported in the literature.

## 2. Methods

### 2.1. Plant Material

Fruits of *E. jambolana* were procured from the Azadpur Mandi (vegetable market) at Delhi. The identity was made with the help of a botanist using taxonomic rules (voucher specimen no. P- 96/7) and specimen are kept for further references in Botanical Garden, Kolkata, India.

### 2.2. Animals

Male albino rabbits (1–1.5 kg) were obtained from Central Animal House, University College of Medical Sciences (UCMS), Delhi, India. Rabbits were maintained on a commercial diet (Hindustan Lever Ltd, Mumbai, India) and water *ad libitum*. They were housed at a temperature of 22 ± 2°C with a schedule of 12 h light and 12 h dark cycle. They were acclimatized to laboratory conditions at least for 1 week before carrying out any experimental work. The experimental protocol for the present study was approved by Institutional Animal Ethical Committee (IAEC) of University College of Medical Sciences, Delhi, India.

### 2.3. Induction of Experimental Diabetes

Experimental diabetes was induced in rabbits with alloxan (80 mg kg^−1^ bodyweight, Sigma Chemicals, USA) dissolved in 0.1 M citrate buffer (pH 4.0), injected intravenously to overnight fasted rabbits through their marginal ear vein [29]. The animals were monitored for plasma glucose levels at weekly intervals for a month. After one month, the rabbits showing stabilized diabetes with fasting blood glucose (FBG) value between 120 and 250 mg dL^−1^ and having abnormal glucose tolerance in glucose tolerance test (GTT) were considered as MD and those with FBG value of 250 mg dL^−1^ or above as SD. Rabbits that did not show any increase in FBG levels even initially after alloxan injection were considered as totally resistant and excluded from studies.

### 2.4. Preparation of the Ethanolic Extract from Seeds of *E. Jambolana* Fruits

One kilogram fruits were first purchased from local market. Fruits were washed well with plenty of tap water and pulp was separated manually from the seeds. Seeds were washed thoroughly to remove all the traces of pulp from the seeds. Seeds of *E. jambolana* were dried at room temperature. Dried seeds were ground in an electric grinder to obtain coarse seed powder. One hundred gram seed powder was suspended in distilled water (250 mL) and allowed to stand at 4°C. It was then filtered through several layers of muslin cloth and filtrate (water extract) was discarded. The residue was then extracted with ethanol (80%) and allowed to stand for 48 h at 4°C. Finally, it was extracted with 95% ethanol. The extract was filtered through seven to eight layers of muslin cloth and then lyophilized at reduced pressure. The yield of alcoholic extract of *E. jambolana* seeds was 1.5 g 100 g^−1^ of dried powdered seeds.

### 2.5. Purification of Antihyperglycemic Component from Ethanolic Extract of Seeds

The ethanolic extract showed potent antihyperglycemic activity [23], it was subjected to purification via silica gel (60–120 mesh) column chromatography. Fractions were eluted with 95% ethanol. Four fractions were eluted. Out of four only one fraction (S III) showed antihyperglycemic activity. Therefore, S III was further purified via sephadex LH 20 column chromatography (LH). It showed the presence of three fractions which were designated as LH I, LH II and LH III. Activities of these three were tested in alloxan-induced MD and SD rabbits. Dose of the fractions was calculated by evaporating a known volume to dryness.

### 2.6. Assessment of Antihyperglycemic Activity

Antihyperglycemic activity of LH I, LH II and LH III at a dose of 10 mg kg^−1^ body weight were tested in both MD and SD rabbits. The rabbits were divided into three groups (five animals in each group). Group I served as healthy control. Group II and III served as MD and SD, respectively. Both Group II and III were further divided into diabetic control rabbits (subgroup A), diabetic rabbits given LH I (subgroup B), diabetic rabbits given LH II (subgroup C), diabetic rabbits given LH III (subgroup D).

Antihyperglycemic activity was assessed by fall in FBG after 90 min of drug administration and reduction in blood glucose tolerance test in MD rabbits and by fall in fasting blood glucose only in SD rabbits (animals die if glucose tolerance test is performed in severely diabetic rabbits).

LH II was found to possess potent anti hyperglycemic activity (Table 1) and its homogeneity was tested via HPLC employing GPC column (GPC, KF-803, 8 × 300 mm 10 × length). LH II was eluted with 0.05 M tris buffer, pH 7.2 and monitored by UV detector at wavelength 254 nm (Instrument Waters HPLC model 510). Single peak was observed at 22.11 min after sample was injected, suggesting that LH II was almost homogenous. Phytochemical investigation of LH II via NMR, 2D COSY and HMQC showed the presence of saturated fatty acid, Δ^5^ lipid and presence of sterol. Further biochemical investigations to know the exact mechanism of action were done with LHII (10 mg kg^−1^ body weight (bw)). The duration of treatment was 7 days for MD and 15 days for SD rabbits.

### 2.7. Sample Collection

Fasting blood samples were taken before and after treatment in both MD (7-day treatment) and SD (15-day treatment) rabbits. Samples were withdrawn from overnight fasted animals. Blood was collected in tubes containing sodium fluoride and potassium oxalate for estimation of blood glucose. The whole blood, collected in EDTA vials, was used to measure glycosylated hemoglobin (GHb).

In order to know whether the LH II acts by stimulating the release of insulin, plasma insulin was measured in MD and SD rabbits. *In vitro* studies on the release of insulin from the islets of Langerhans were also performed in diabetic rabbits. Insulin levels were estimated using enzyme-linked immunosorbent assay kits (Boehringer Manheim, Germany).

At the end of the study, the rabbits were sacrificed, liver and muscles tissues dissected for glycogen estimation. Activities of key enzymes of carbohydrate metabolism, that is, glucokinase, phosphofructokinase, glucose-6-phosphatase and fructose-1,6-bisphosphatase were also measured in hepatic tissues to know extrapancreatic effect.

### 2.8. Biochemical and Enzymatic Estimations

Blood glucose was estimated by glucose oxidase method [30]. HbA1c was estimated by the method of Goldstein et al. [31]. Islets of Langerhans were isolated as describe by our earlier studies [32]. Plasma insulin was measured by the method of Burgi et al. [33]. Key enzymes of glycolysis, that is, glucokinase [34], phosphofructokinase [35] and of gluconeogenesis, that is, glucose-6-phosphatase [36] and fructose-1,6-bisphosphatase [37] were estimated by already standardized protocols. Tissue glycogen was assayed as described by Carroll et al. [38].

### 2.9. Toxicity Studies

To evaluate the toxicity of high dose of the LH II, three groups of fasted healthy rats (five animals per group) were administered orally graded doses of LH II (up to a dosage of 5, 10 and 15 times of the effective dose, that is, 10 mg kg^−1^ bw) and one group was taken as control given distilled water. The animals were observed for 1 h continuously and then hourly for 4 h and finally after every 24 h up to 30 days for any gross behavioral changes or mortality, if happens. Liver function tests such as serum glutamate phosphotransferase (SGPT) and alkaline phosphatase (ALP) as well as kidney function tests such as urea and creatinine were performed in serum at the end of the study using standard methods.

### 2.10. Statistical Analysis

Values were expressed as the mean ± SEM for five animals in each group. Statistical analysis was done by using repeated measure analysis of variance (ANOVA) and one-way ANOVA followed by Tukey's multiple comparison test at 5% level of significance.

## 3. Results

### 3.1. Glucose Levels: One-day Treatment

As shown in Table 1, LH II (at a dose of 10 mg kg^−1^ bw) was found to be more potent in reducing fasting blood glucose (21.2%) within 90 min and improving peak blood glucose (27% fall) during glucose tolerance test in mild diabetic rabbits. However there was 28.6% fall in FBG of SD rabbits. Hence, further biochemical studies were carried out with LH II only at a dose of 10 mg kg^−1^.

### 3.2. FBG, OGTT and GHb

**Figure 1:**
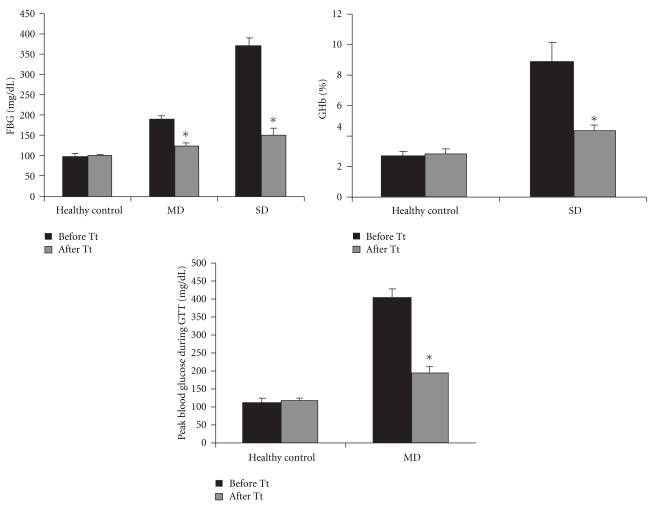
Antidiabetic effect of LH II (10 mg kg^−1^) in mild diabetic and severe diabetic rabbits. Values are mean ± SEM for five animals in each group. *P < .001 versus before treatment.

As shown in Figure 1, LH II resulted 35.6% fall in FBG and 51.3% fall in peak blood glucose during GTT in MD after 7 days treatment and in SD rabbits, 59.6% fall in FBG and 50.5% fall in HbA1c after 15 days treatment.

### 3.3. Insulin Levels: Both In Vivo and In Vitro

**Figure 2:**
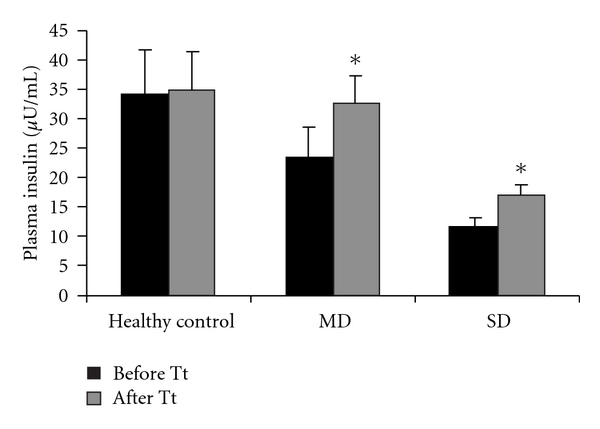
Effect of LH II on Plasma insulin in mild diabetic and severely diabetic rabbits. Values are mean ± SEM for five animals in each group. *P < .001 versus before treatment.

Oral administration of LH II, resulted significant increase in plasma insulin levels, that is, 38.4% in MD and 44.0% in SD (Figure 2). *In vitro* studies conducted in diabetic rabbits resulted in 3-fold increase of insulin release (Table 2).

### 3.4. Hepatic Enzymes

**Figure 3:**
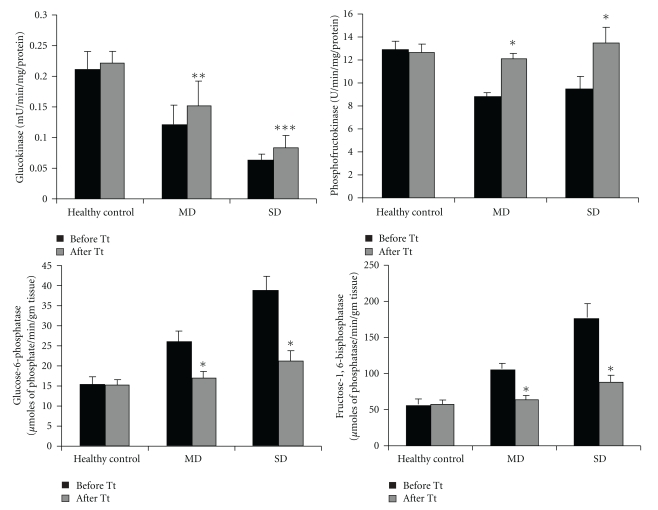
Effect of LH II on carbohydrate metabolizing enzymes in mild diabetic and severely diabetic rabbits. Values are mean ± SEM for five animals in each group. *P < .001, **P < .001, ***P < .005 versus before treatment.

Figure 3 shows glucokinase, phosphofructokinase, glucose-6-phosphatase and fructose-1,6-bis phosphatase activities in the livers of control and diabetic rabbits.

In diabetic rabbits, activities of key enzymes of glycolysis decrease and key enzymes of gluconeogenesis increase. After oral administration of LH II, these activities were partially restored to near normal levels in both MD and SD rabbits.

### 3.5. Total Lipids and Glycogen Content

**Figure 4:**
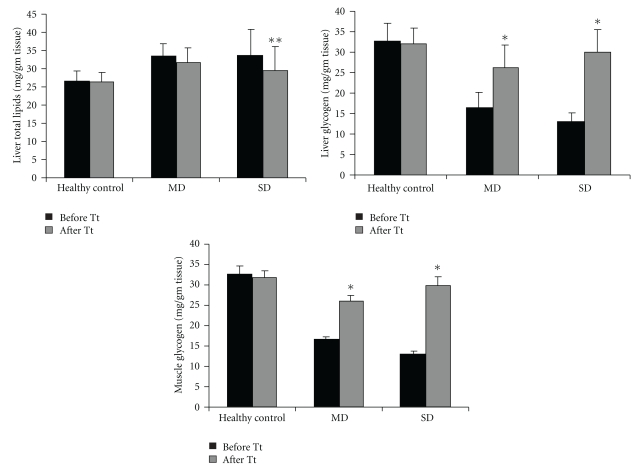
Effect of LH II on total lipids and glycogen content in mild diabetic and severely diabetic rabbits. Values are mean ± SEM for five animals in each group. *P < .001, **P < .001 versus before treatment.

Figure 4 shows, the levels of total lipids in liver and glycogen in both liver and muscle. A increase in total lipids was found in diabetic rabbits when compared with control. The administration of LH II (10 mg kg^−1^) resulted in significant reduction in total lipids in both MD (10.7% fall) and SD (11.5%) and significant increase in liver and muscle glycogen content (*P* <  .001) in both MD and SD rabbits.

### 3.6. Toxicity Studies

Toxicity studies revealed that the administration of graded doses of LH II (up to a dosage of 5, 10 and 15 times of 10 mg kg^−1^) produced no adverse effect on the general behavior or appearance of the animals and all the rats survived during the whole experimental period. No significant change was observed in the levels of SGPT and ALP in the treated groups when compared with control group. The levels of urea and creatinine were also not significantly changed in treated animals compared to control (Table 3).

## 4. Discussion

Hypoglycemic and hypolipidemic effect of ethanolic extract of seeds of *E. jambolana* in normal and alloxan induced (80 mg kg^−1^) MD and SD rabbits had already been shown [23]. The main goal of present study was to purify active principle from ethanolic seed extract and find its exact mechanism of action in respect of carbohydrate metabolism.

Studies conducted by us using graded doses of LH II for various periods revealed the non-toxic nature of the LH II. Rabbits that were administered LH II did not show any drug induced toxic symptoms, even after 4 weeks of the experimental period. Thus, the LH II was found to be safe for further biological studies.

As shown in Table 1, LH II showed significant antihyperglycemic activity (*P* < .001). MD and SD failed to achieve euglycemia but caused a significant (*P* < .001) reduction in glucose levels compared to their initials values. Although, the percent fall in blood glucose was found to be significant in MD rabbits which had functional pancreatic *β*-cells, significant fall in SD rabbits suggested that the LH II did not necessarily require the presence of functional pancreatic *β*-cells for its favorable action. It means that it could act in a variety of diabetic conditions with or without functioning pancreatic *β*-cells. During diabetes, the excess of glucose present in the blood reacts with Hb to form GHb. The rate of glycosylation is proportional to the concentration of blood sugar at the peak of the glucose tolerance curve, correlates with glycosylation [39] and, with an improvement in glycemic control, GHb also decrease. Hence, estimation of glycosylation of Hb is a well-accepted parameter used in the management and prognosis of the disease [40]. In the present study, administration of the LH II tended to bring the altered level of GHb toward normal range in both MD and SD rabbits. This was due to the improved glycemic control produced by the LH II.

The loss of body weight is one of the threats associated with diabetes mellitus. Treatment with the LH II prevented this loss and rather resulted in increase of body weight in MD and SD rabbits.

Antihyperglycemic action of LH II may be due to increased utilization of glucose in the liver by glycogen synthesis, decreased degradation of glycogen to give blood sugar and also to decrease gluconeogenesis [41]. Another possible mechanism of lowering blood glucose levels may be by potentiation of the insulin effect by increasing either the pancreatic secretion of insulin from *β*-cells of the islets of Langerhan's or the responsiveness to insulin.

Diabetes mellitus cause a disturbance in the uptake of glucose, as well as glucose metabolism. The liver plays an important role in the maintenance of blood glucose levels by regulating its metabolism. The activity of glucokinase, which brings about the first phosphorylation step of glucose metabolism and phosphofrutokinase was significantly reduced in diabetic rabbits. This could be the reason for the diminished utilization of glucose in the system and the increased amount of glucose in the blood [42]. Increased glucokinase activity was observed in the alloxan induced diabetic rabbits treated with LH II, which would have resulted in the activation of glycolysis, which, in turn, increased the utilization of glucose by restoring insulin secretion in the treated rabbits.

Glucose-6-phosphatase is one of the important regulatory enzyme of the gluconeogenic pathway [43]. Fructose-1,6-bisphosphatase is the key enzyme catalyzing the rate limiting steps of fructose-1,6-bisphosphate to fructose-6-phosphate. The activities of glucose-6-phosphatase and fructose-1,6-bisphosphatase are increased in the liver in diabetic subject [44]. This results in a decrease in the glycolytic flux. Under normal condition, insulin functions as a suppressor of gluconeogenic enzymes [45]. The increased activities of these gluconeogenic enzymes in diabetic rabbits were restored to near normal levels, after administration of LH II. The possible mechanism by which LH II brought about the normalization of enzymes might be by potentiation of insulin release from *β*-cells of the islets of Langerhan's or its release from the bound form.

Glycogen is the primary intracellular storage form of glucose and its levels in various tissues are a direct reflection of insulin activity, because insulin promotes glycogen deposition by stimulating glycogen synthase and inhibiting glycogen phosphorylase [46]. Diabetes mellitus is associated with a marked decrease in the level of liver glycogen [47, 48]. Our data also showed significant decrease in hepatic and skeletal glycogen content in diabetic control. Oral administration of LH II to diabetic rabbits showed an increase in glycogen content of liver and skeletal muscles; this might be due to decreased glycogen phosphorylase activity and increased glycogen synthase activity.

Our findings suggested that LH II could prove to be an excellent agent in the control of experimental diabetes. Therefore, the mechanism of action of LH II appeared to be both pancreatic [19] and extra-pancreatic as *E. jambolana* is reported to inhibit insulinase activity in both liver and kidney [20]. The results of present study provides impetus for further molecular and mechanistic studies on the therapeutic action of LH II, before it can be administered as possible insulin replacement or adjuvant in the management of diabetes mellitus.

## Funding

Director-General, Indian Council of Medical research, New Delhi, India.

## Figures and Tables

**Figure 5:**
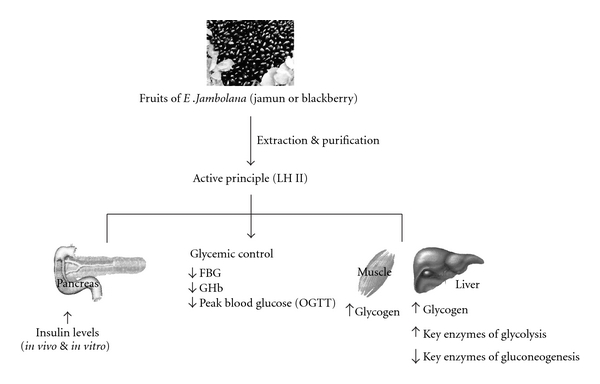
Therapeutic approach of active principle (LH II) purified from seeds of Eugenia jambolana on altered carbohydrate metabolism.

**Table 1:** Antihyperglycemic activity of LH I, LH II and LH III in mild (MD) and severe diabetic (SD) rabbits.

Group	Dose (mg kg^−1^)	FBG (percentage change) FBG after 90 min	Peak glucose during GTT
Mild Diabetic			
LH I	10	7 (↑)	5 (↑)
LH II	10	21.2 (↓)	27.0 (↓)
LH III	10	10.0 (↓)	No change
Severe Diabetic			
LH I	10	9 (↑)	—
LH II	10	28.6 (↓)	—
LH III	10	12.0 (↓)	—

**Table 2:** Effect of LH II on insulin release from isolated islets of Langerhans of diabetic rabbits

Group	Dose	Insulin Release (*μ* U g^−1^ islet)	Fold increase
Diabetic	—	18195 ± 398.0	—
Diabetic + LH II	10 mg kg^−1^	56250 ± 153.6*	3-fold

Values are mean ± SEM for five animals in each group. *P < .001 versus diabetic without treatment.

**Table 3:** Effect on liver and kidney function tests during oral toxicity studies of active principle (LH II) isolated from ethanolic extract of seeds in normal rabbits (1 month treatment).

Parameter	Healthy control	Treated 5 times of effective dose	Treated 10 times of effective dose	Treated 15 times of effective dose
Serum Bilirubin (mg dL^−1^)	0.45 ± 0.02	0.44 ± 0.03	0.44 ± 0.02	0.44 ± 0.02
SGPT (IU L^−1^)	11.2 ± 1.1	12.1 ± 1.3	11.5 ± 0.9	11.5 ± 0.9
Serum ALP (U L^−1^)	33.0 ± 2.4	33.2 ± 3.1	33.0 ± 2.8	33.0 ± 2.8
Serum protein (g dL^−1^)	8.0 ± 0.9	7.9 ± 0.6	8.0 ± 0.6	8.0 ± 0.2
Blood urea (mg dL^−1^)	15.0 ± 1.3	15.0 ± 1.7	15.9 ± 1.5	15.4 ± 1.8
Serum Creatinine (mg dL^−1^)	1.3 ± 0.1	1.4 ± 0.3	1.4 ± 0.1	1.4 ± 0.2

Values are mean ± SEM for five animals in each group. No significant difference between control and treated groups.
